# From disease management to prevention, hip prosthesis joint infections in the past 20 years: a global research trends and top 10 cited articles analysis

**DOI:** 10.3389/fsurg.2024.1448049

**Published:** 2025-01-07

**Authors:** Fei-Long Li, Xing-Yu Qi, Jin-Lun Chen, Yi-Rong Zeng

**Affiliations:** ^1^ The First Clinical Medical College, Guangzhou University of Chinese Medicine, Guangzhou, Baiyun, China; ^2^ Department of Orthopaedics (Joint Center), The Third Affiliated Hospital of Guangzhou University of Chinese Medicine, Guangzhou, Liwan, China; ^3^ Department of Orthopaedics, The First Affiliated Hospital of Guangzhou University of Chinese Medicine, Guangzhou, Guangdong, China

**Keywords:** hip, prosthesis joint infections, bibliometric analysis, research trends, highly cited articles background

## Abstract

**Background:**

There are few literatures comprehensively analyzed the global research in hip prosthesis joint infections (HPJI). We aim to clarify the global research trends and analysis the top 10 cited articles in the HPJI field.

**Methods:**

We identified the core collection of articles/reviews in the HPJI field from 2001 to 2021 through the Web of Science Core Collection (WOSCC). VOSviewer and online bibliometric tool were used to conduct the visualized and knowledge maps. Annual trends of publications, research hotspots and the top 10 cited articles were analyzed.

**Results:**

A total of 5,477 publications were finally included. Generally, an increasing trend was observed in the number of publications from 2001 to 2021. The authors, journals and institutions with largest number of publications all belong to the USA. Co-occurrence analysis of keywords showed that surgical techniques, risk factors, revision surgery strategy, epidemiology, diagnoses and prevention were the 6 major research directions. Total hip arthroplasty, replacement, outcomes, risk factors and diagnosis were the keywords that occurred most frequently. The top 10 cited articles were all published in Journal Citation Reports (JCR) Q1 journals, providing valuable reference value from the perspectives of clinical guidelines, perioperative management, causes and diagnostic methods of infection, epidemiological investigation, risk factors and prognostic analysis.

**Conclusions:**

The number of publications in HPJI field had been on the rise over the past 20 years, from disease management to prevention. An intensive reading of the top 10 cited articles is beneficial to understand the focus of HPJI research comprehensively.

## Background

As the aging of population, more patients with end-stage hip disease are undergoing total hip arthroplasty (THA) (1). THA is a mature procedure with well-defined clinical effect, however, the risk of failure is still existing with a certain probability (2). Hip prosthesis joint infections (HPJI) represents the second most frequent, but also the most important complication of total joint arthroplasty after aseptic loosening. Once it occurred, it is a huge challenge for both orthopedists and patients. HPJI could lead to hip dysfunction and thus decreasing the quality of life, and even causing complications of other organs (3). Revision surgery gradually becomes a huge economic burden with the cost of $45,000 to $160,000 per case (4). Therefore, the scientific researchers from various countries are sparing no effort to deepen the study of this disease. Over the past 2 decades, scholars have carried out high-level studies on risk factor analysis, epidemiological investigation, optimization of diagnostic methods, upgrading of treatment technology, standardization of preventive measures and other aspects (5–10). This undoubtedly provided valuable clinical evidence for scientific researchers. However, since these articles are scattered, it is difficult to grasp its development trend and hotspots. At the same time, some inferior quality articles can also be confusing. Therefore, it is necessary to use new methods to have an insight into the publications in HPJI field, analyze the global research trends and highly cited articles, and help scientific researchers better grasp the trends and hot spots.

Bibliometric is a new interdisciplinary research method in recent years, which integrates statistics and visualization technology and helps to understand the research frontier, development trend and hot spot of a discipline (11). Bibliometric have been used in orthopedic studies such as hip fractures, knee osteoarthritis and shoulder instability. And with those valuable studies, orthopedists can have a direct understanding of the development trend of these fields (12–14). Bibliometrics is a good literature review and analysis method, which can help orthopedic scholars to understand the dynamic changes of research hotspots, focuses and research trends in their own field over a period of time. Therefore, the aim of this study is to analyze the global research trends and the top 10 cited articles in HPJI field through bibliometric. In addition, we want to summarize the hotspots and directions of HPJI research, hoping to provide more useful references for future research.

## Methods

### Data source and retrieval strategies

**Figure 1:**
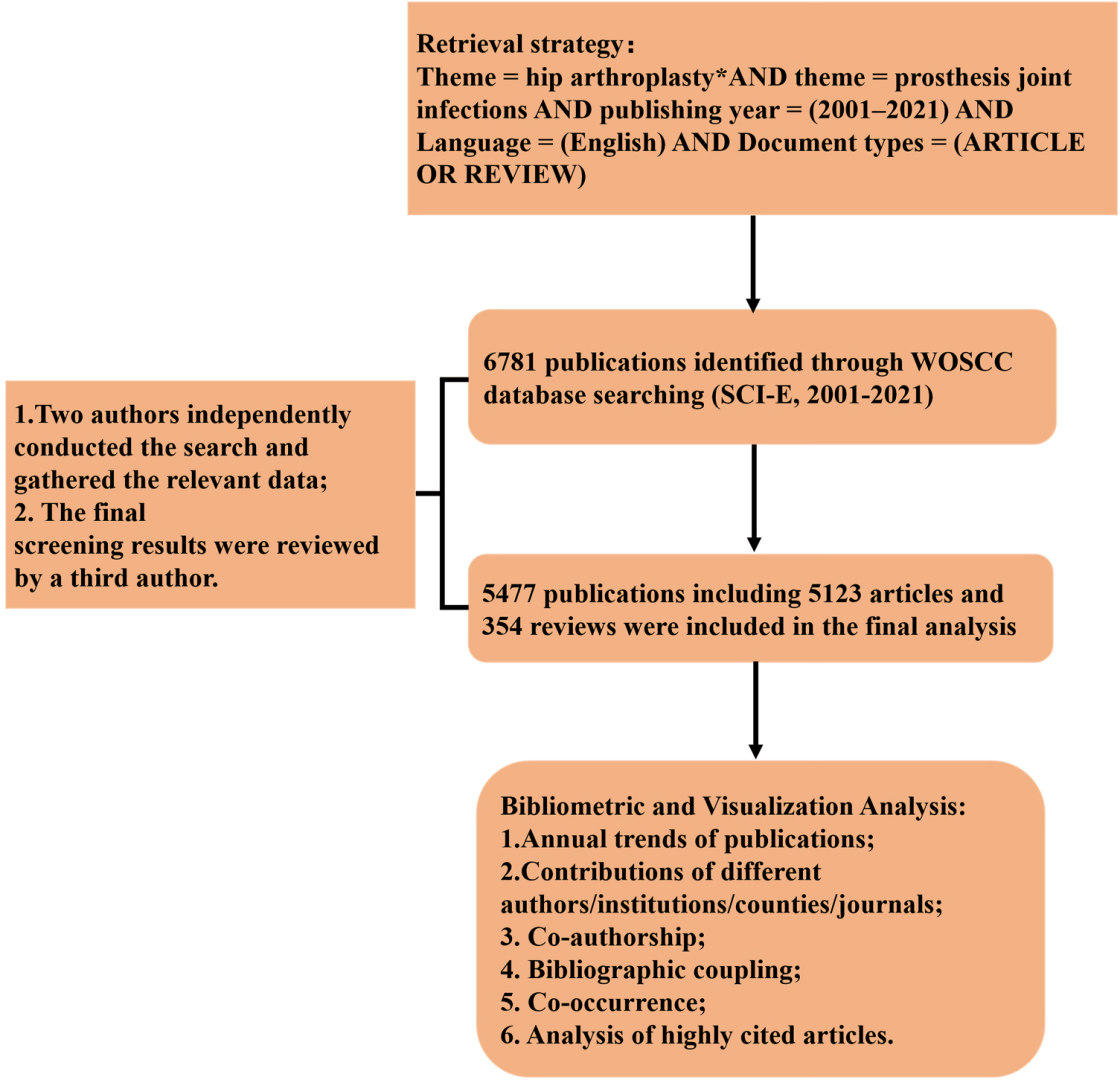
Flowchart for the selection of articles/review included in the study.

We retrieved the publications in HPJI field from the Web of Science Core Collection (WOSCC) (www.webofscience.com), using the following retrieval strategy: theme = hip arthroplasty*AND theme = infections* AND publishing year = (2001–2021) AND Language = (English) AND Document types = (ARTICLE OR REVIEW). After eliminating the publications irrelevant to the research subject, we download the core information of the publications conforming to the retrieval strategies from WOSCC and save it in TXT format, including the titles, authors, abstracts, keywords and references (Figure 1).

### Data extraction and descriptive analysis

The TXT files of 5,477 publications finally included in this study were imported into Microsoft Excel 2019 (Microsoft Corporation, Redmond, Washington, USA) for further analysis. We identified the number of publications published in different years, the number of publications, average citation per item (ACI) and H-indexes of different authors, institutions, countries and journals were also extracted, respectively (The above indicators are subject to change over time. The data collected in this study are real-time data from WOSCC on January 15, 2022). The contributions of the top 10 authors/institutions/counties/journals were manifested through the number of publications, ACI and H-index (15). Microsoft Excel 2019 was used to create bar charts for the above data.

### Bibliometric and visualized analysis

We used VOSviewer (Version1.6.8, Centre for Science and Technology Studies, Leiden University, The Netherlands) and the online bibliometric tool (https://bibliometric.com/app_v0) to complete the bibliometric and visualized analysis. The annual trends of publications were performed on the online bibliometric tool, the network visualization map of co-authorship, bibliographic coupling and co-occurrence were performed on VOSviewer. Different nodes in the network visualization map indicated various parameters, such as authors, institutions, countries, journals, literatures and keywords. The size of the nodes represents the total link strength (TLS) in the co-authorship analysis, represents the frequency of citations in the bibliographic coupling and represents the frequency of occurrence in the co-occurrence analysis, respectively. The thickness of the line connecting different nodes indicates the relationship between the two nodes.

### Analysis of the top 10 cited articles

In WOSCC, the top 10 cited articles were identified through citation frequency analysis. After selecting the top 10 cited articles, we analyzed and summarized their source journals, impact factor (IF), JCR, the number of citations and focus point.

## Results

### Global publications from 2001 to 2021

**Figure 2:**
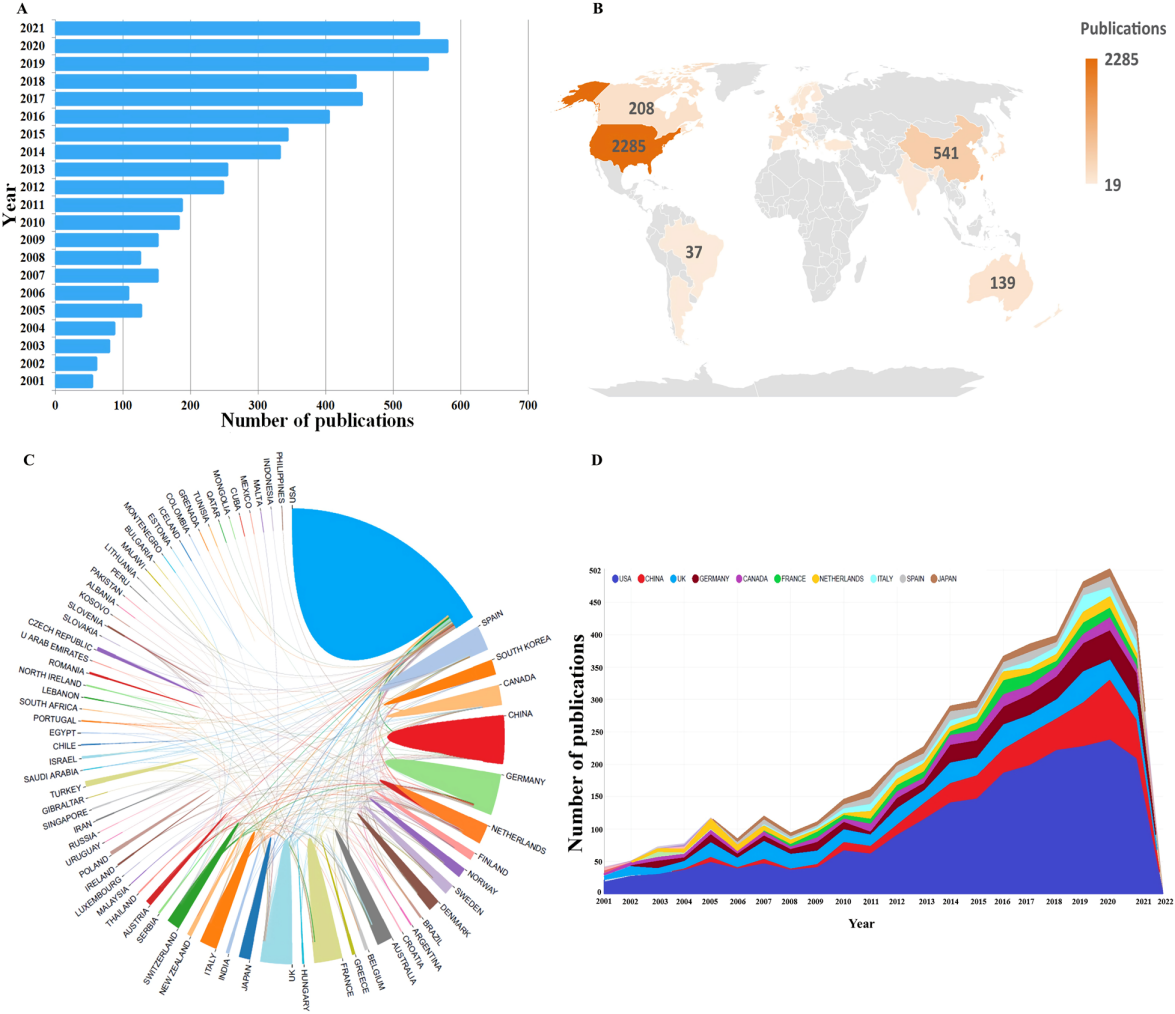
Overview of global publications and trends in HPJI field. **(A)** The number of publications published annually in the HPJI field. **(B)** A world map showing the number of global publications in HPJI field from 2001 to 2021. Different colors on the map represent the total number of posts from different countries, the number of publications of Taiwan, Hong Kong and Macao were included in China. **(C)** The cooperation map of countries/regions involved in HPJI research. The larger the area of the colored area, the larger the number of publications. The thicker the lines connecting different areas, the closer the cooperative relationship. **(D)** The publication trends of the top 10 active countries in HPJI field from 2001 to 2021. Annual trends in the number of publications published in different countries in different years are shown in the figure as widths of different color lines.

After strict extraction according to the criteria, a total of 5,477 publications from 2001 to 2021 in HPJI field, including 5,123 articles and 354 reviews, were finally identified (Figure 1). Overall, there has been an increase in global publications over the past 20 years, the growth rate accelerated significantly after 2011, with 200 articles published every year (Figure 2A). There is an uneven distribution of the publications, mainly in the North America, Europe and East Asia, followed by the Oceania and South America (Figure 2B). The international collaboration analysis indicated that the USA collaborated most closely with Germany, UK, Canada and China (Figure 2C). The annual transformative trends of the top 10 countries by the number of publications from 2001 to 2021 also showed an overall upward trend (Figure 2D).

### Contributions of the Top 10 authors

**Figure 3:**
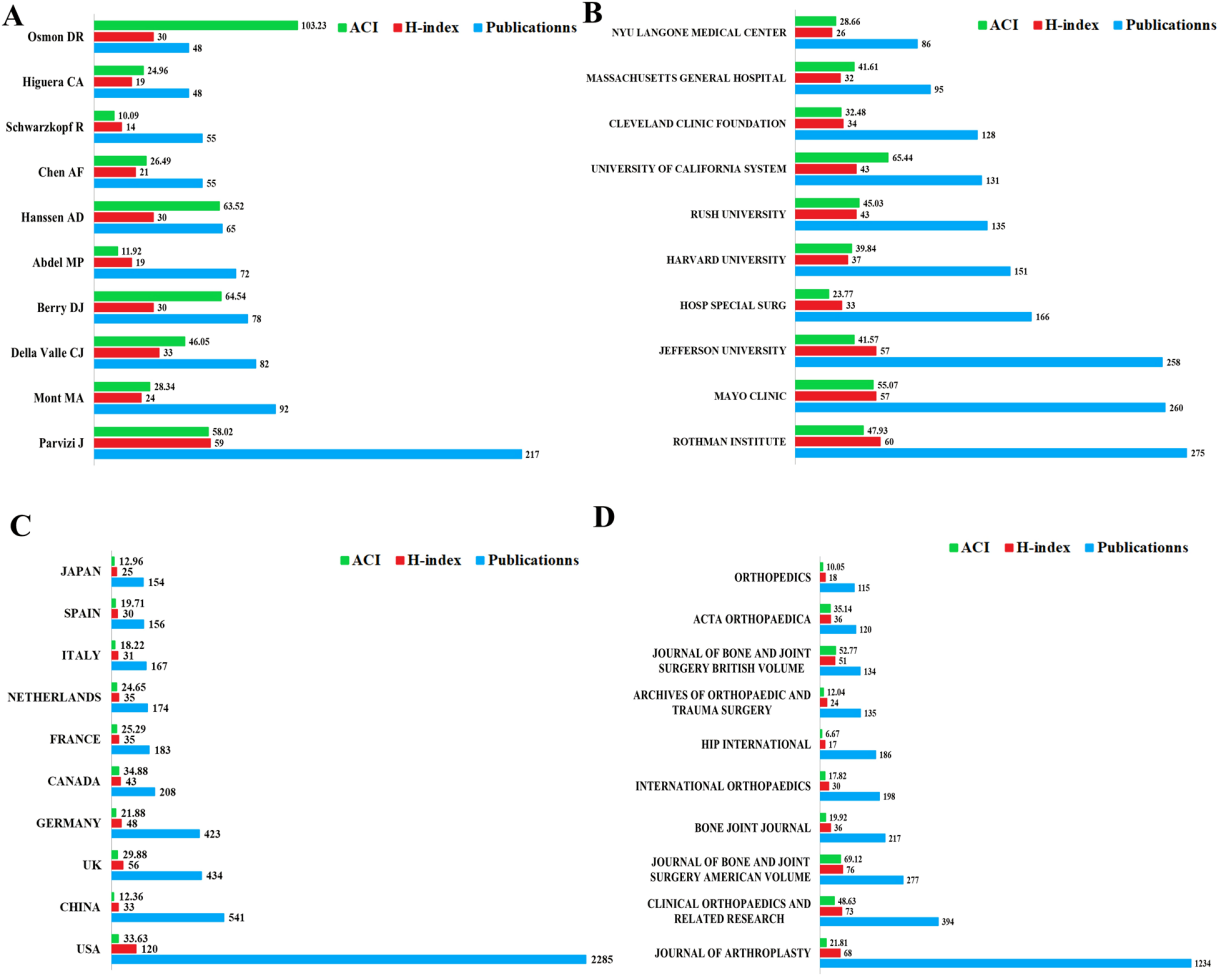
The top 10 authors institutions/countries/journals contributed to research publications in HPJI field. **(A)** The top 10 authors contributed to research publications in HPJI field. **(B)** The top 10 institutions contributed to research publications in HPJI field. **(C)** The top 10 countries contributed to research publications in HPJI field, the number of publications of Taiwan, Hong Kong and Macao were included in China. **(D)** The top 10 journals contributed to research publications in HPJI field. ACI, average citation per item.

As presented in Figure 3A, Parvizi J had the largest number of publications (*n* = 217) and highest H index (*n* = 59), while Osmon DR had the highest ACI (*n* = 103.23). In terms of the number of publications, the top 10 authors, in descending order, were Parvizi J (*n* = 217), Mont MA (*n* = 92), Della Valle CJ (*n* = 82), Berry DJ (*n* = 78), Abdel MP (*n* = 72), Hanssen AD (*n* = 65), Chen AF (*n* = 55), Schwarzkopf R (*n* = 55), Higuera CA (*n* = 4) and Osmon DR (*n* = 48). As for the H-index, the top 10 authors were as followed: Parvizi J (*n* = 59), Della Valle CJ (*n* = 33), Berry DJ (*n* = 30), Hanssen AD (*n* = 30), Osmon DR (*n* = 30), Mont MA (*n* = 24), Chen AF (*n* = 21), Abdel MP (*n* = 19), Higuera CA (*n* = 19) and Schwarzkopf R (*n* = 14). In terms of the ACI, the top 10 authors were as followed: Osmon DR (*n* = 103.23), Berry DJ (*n* = 64.54), Hanssen AD (*n* = 63.52), Parvizi J (*n* = 58.02), Della Valle CJ (*n* = 46.05), Mont MA (*n* = 28.34), Chen AF (*n* = 26.49), Higuera CA (*n* = 24.96), Abdel MP (*n* = 11.92) and Schwarzkopf R (*n* = 10.09).

### Contributions of the Top 10 institutions

As presented in Figure 3B, the top 10 institutions contributed to research publications in HPJI all belong to the USA, Rothman Institute (Thomas Jefferson University Hospitals, Philadelphia, PA, USA) had the largest number of publications (*n* = 275) and the highest H-index (*n* = 60), while the University of California System (University of California, CA, USA) had the highest ACI (*n* = 65.44). The number of publications of the top 10 institutions ranged from 86 to 275, the H-index ranged from 26 to 60, and the ACI ranged from 23.77 to 65.44, respectively.

### Contributions of the Top 10 countries

As presented in Figure 3C, the USA had the largest number of publications (*n* = 2,285, 41.7%) and the highest H index (*n* = 120), while Canada had the highest ACI (*n* = 34.88). In terms of the number of publications, the top 10 countries, in descending order, are the USA, China, UK, Germany, Canada, France, Netherlands, Italy, Spain and Japan, ranged from 2,285 (41.7%) to 154 (2.8%). As for the H-index, the order is as followed: the USA, UK, Germany, Canada, France, Netherlands, China, Italy, Spain and Japan, ranged from 120 to 25. As for the ACI, the top 10 countries are as followed: Canada, the USA, UK, France, Netherlands, Germany, Spain, Italy, Japan and China, ranged from 34.88 to 12.36.

### Contributions of the Top 10 journals

As presented in Figure 3D, the Journal of Arthroplasty had the largest number of publications (*n* = 1,234, 22.5%), the Journal of Bone and Joint Surgery American Volume had the highest H-index (*n* = 76) and ACI (*n* = 69.12). The number of publications of the top 10 journals ranged from 115 (2.0%) to 1,234(22.5%), the H-index ranged from 17 to 76, and the ACI ranged from 6.67 to 69.12, respectively. The top 10 journals are all specialized in orthopedics.

### Co-authorship analysis

**Figure 4:**
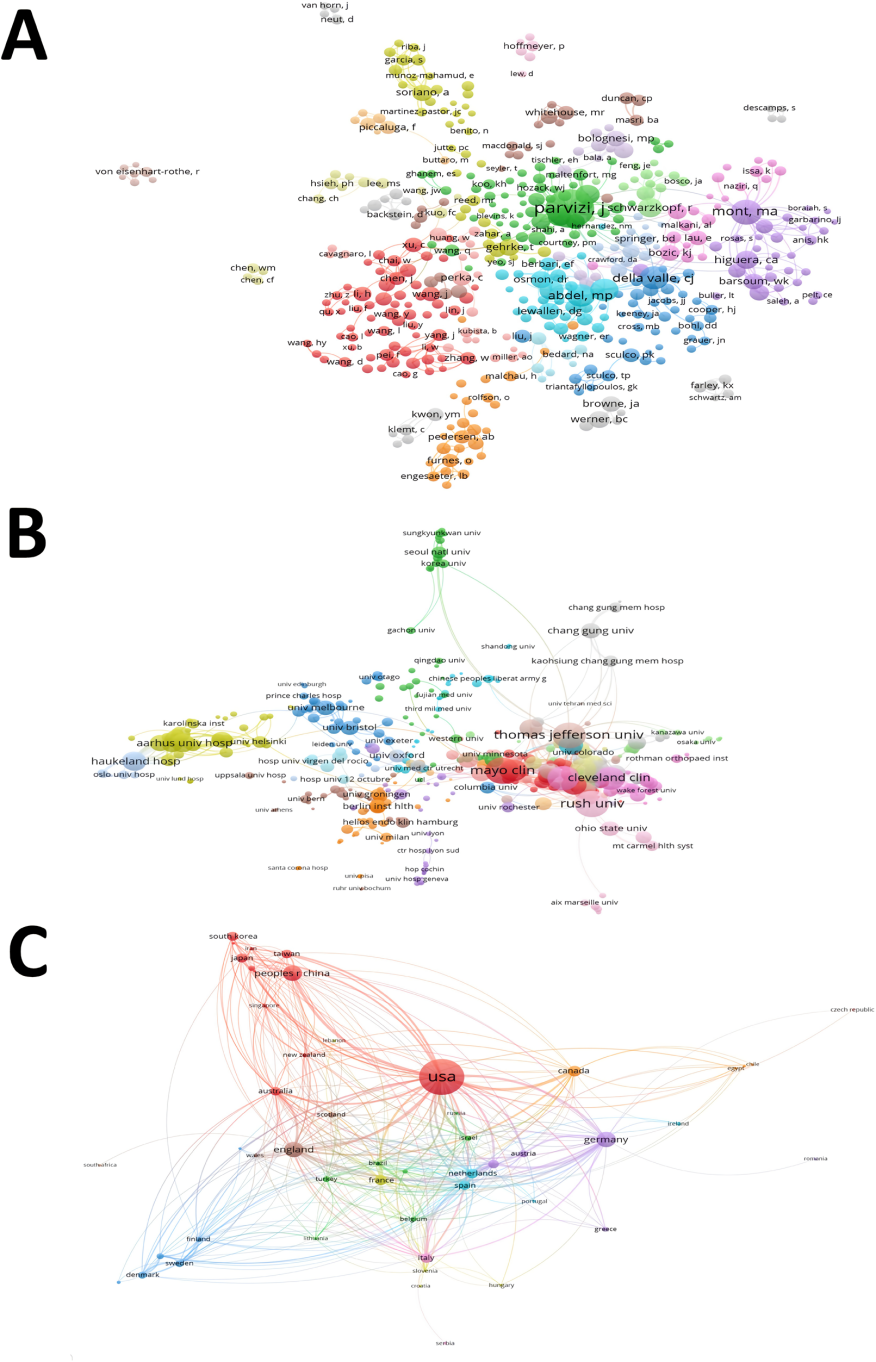
Co-authorship analysis of global research about HPJI. **(A)** Mapping of the 1,002 authors co-authorship analysis on HPJI. **(B)** Mapping of the 467 institutions co-authorship analysis on HPJI. **(C)** Mapping of the 50 countries co-authorship analysis on HPJI. Each node represents a different author/organization/country, the size of nodes represents the frequency of cooperation and the thicker the line between the two nodes, the closer the collaboration between them.

We set the minimum number of publications of 5 as criteria when performing co-authorship analysis. Four hundred and seventy-seven authors that had co-authorship with others were finally included in the analysis (Figure 4A). Parvizi J had the greatest TLS (TLS = 557), followed by Mont MA (TLS = 331), Berry DJ (TLS = 264), Della Valle CJ (TLS = 237), and Abdel MP (TLS = 234). Four hundred and forty-two institutions that had co-authorship with others were finally included in the analysis (Figure 4B). Rush University had the greatest TLS (TLS = 195), followed by Jefferson University (TLS = 187), Mayo Clinic (TLS = 169), Hospital for Special Surgery (TLS = 169), and Cleveland Clinic (TLS = 148). Fifty countries that had co-authorship with others were finally included in the analysis (Figure 4C). The USA had the greatest TLS (TLS = 402), followed by UK (TLS = 182), Germany (TLS = 178), Netherlands (TLS = 114), and France (TLS = 113). The co authorship of the above authors reflects the close cooperation between different scholars in this field, especially International collaborations, from 2001 to 2021, there have been more than 10 consensus/guidelines on PJI developed by experts from countries around the world, and these studies have made important contributions to advancing the diagnosis, management and prevention of HPJI.

### Bibliometric coupling analysis

**Figure 5:**
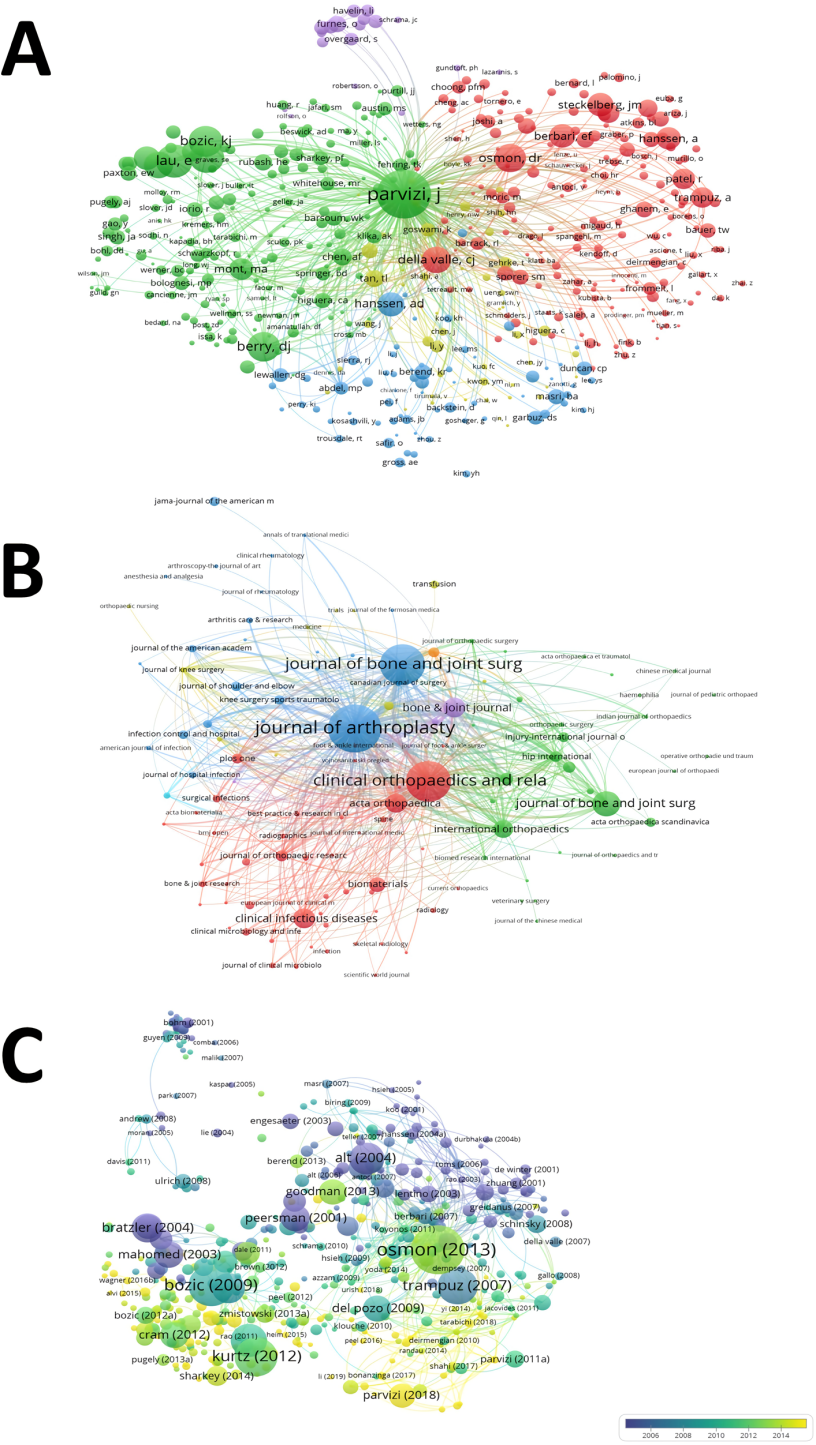
Bibliographic coupling analysis of global research about HPJI. **(A)** Mapping of the 314 authors on HPJI. **(B)** Mapping of the 121 journals on HPJI. **(C)** Mapping of the 5,477 articles/review on HPJI from different years. The line between two nodes represents that two authors/journals/articles had establish a similarity relationship, the thickness of the line indicates the strength of the connection.

As setting the minimum number of citations of 5 as criteria when proceeding bibliometric Coupling Analysis, one thousand and two authors, one hundred and twenty-one journals and 3,809 articles/reviews were finally included. The 5 authors with the highest frequency of citations were as followed (Figure 5A): Parvizi J (citations = 12,273), Osmon DR (citations = 4,041), Della Valle CJ (citations = 3,545), Hanssen AD (Citations = 2,952) and Berbari EF (citations = 2,858). The 5 journals with the highest frequency of citations were as followed (Figure 5B): the Journal of Arthroplasty (citations = 26,905), Clinical Orthopaedics and Related Research (citations = 19,157), Journal of Bone and Joint Surgery American Volume (citations = 19,142), Journal of Bone and Joint Surgery British Volume (citations = 7,069) and Bone Joint Journal (citations = 4,323). The 5 articles/reviews with the highest frequency of citations were as followed (Figure 5C): Osmon DR et al (16) (2013, citations = 1,236), Bozic KJ et al (17) (2009, citations = 980), Kurtz SM et al (18) (2012, citations = 911), Trampuz A et al (19) (2007, citations = 835) and Alt V et al (20) (2003, citations = 686).

### Co-occurrence analysis

**Figure 6:**
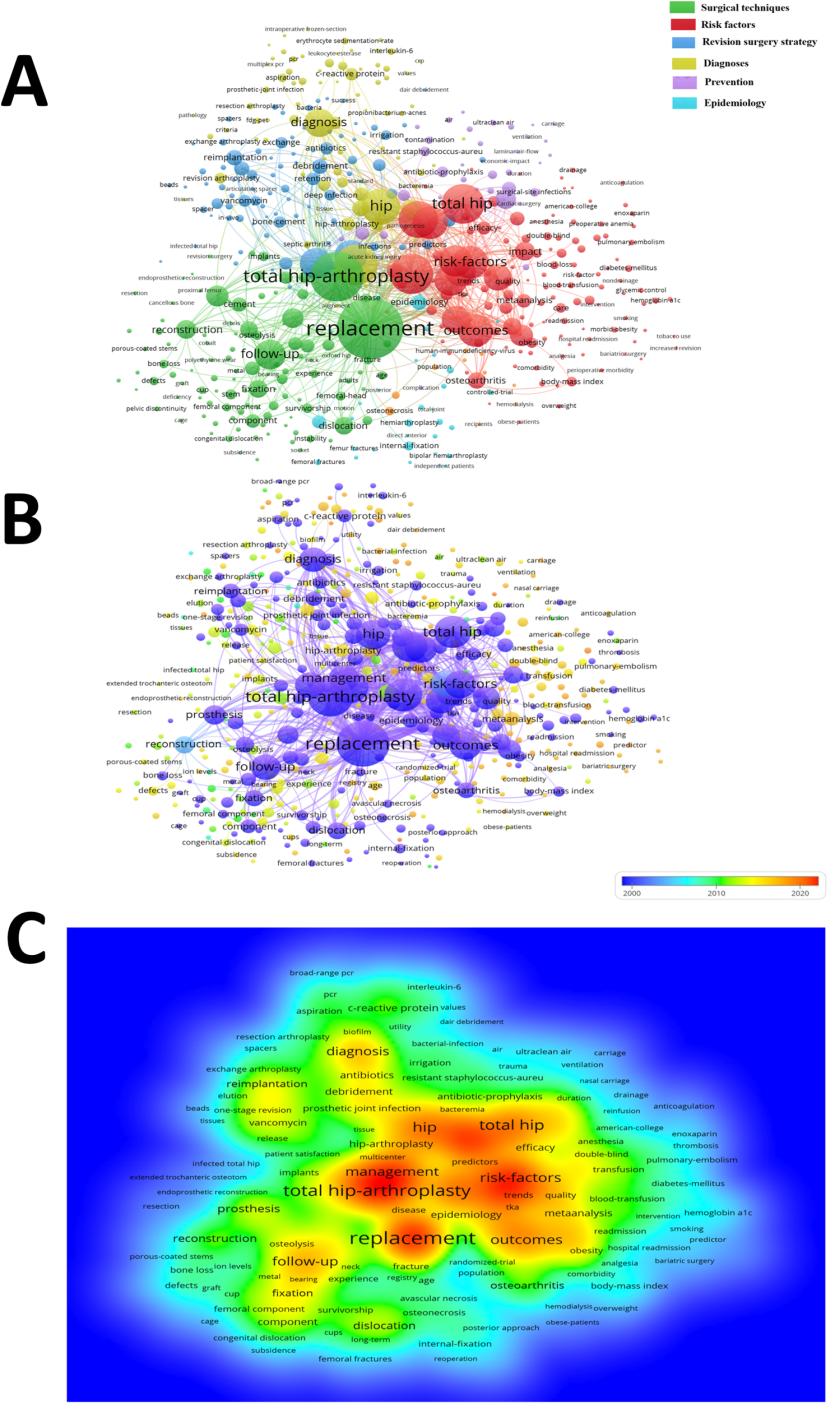
Co-occurrence analysis of global research about HPJI. **(A)** Mapping of keywords in the research on HPJI, the size of the nodes represents the frequency of occurrence, and the keywords were divided into five clusters. **(B)** Chronological distribution of keywords according to the mean frequency of occurrence, keywords in purple appeared earlier than those in yellow and red. **(C)** Distribution of keywords according to the density of occurrence, the depth of the color represents the density of the keywords.

As setting the minimum number of citations of 5 as criteria, 1,049 keywords were finally included in the co-occurrence analysis that divided into 6 clusters: surgical techniques (Green), risk factors (Red), revision surgery strategy (Royal blue), diagnoses (Yellow), prevention (Purple) and epidemiology (Azure) (Figure 6A). The 5 keywords with the highest frequency of occurrence were as followed: replacement (occurrence = 1,542), total hip arthroplasty (occurrence = 1,077), infection (occurrence = 753), arthroplasty (occurrence = 747) and total hip (occurrence = 734). We also categorized the keywords according to the time they appeared in the literature. Most studies focused on hip replacement techniques and infection management from 2001 to 2010. From 2011 to 2021, risk factors analysis, novel diagnostic techniques and indicators became more prominent in HPJI field (Figure 6B). After integration, the keywords with the highest frequency density were total hip arthroplasty, replacement, management and risk factors, respectively (Figure 6C).

### Analysis of the Top 10 cited articles

According to the number of citations, the top 10 cited articles were as presented (Table 1): Osmon DR et al (16) (2013, citations = 1,236), Bozic KJ et al (17) (2009, citations = 980), Kurtz SM et al (18) (2012, citations = 911), Trampuz A et al (19) (2007, citations = 835), Alt V et al. (20)(2003, citations = 686), Kurtz SM et al (21) (2007, 622), Bratzler DW et al (22) (2004, citations = 596), Mahomed NN et al (23) (2003, citations = 534), Del Pozo JL et al (24) (2009, citations = 512), Parvizi J et al (25) (2018, citations = 463). Among these 10 highly cited articles, five were from orthopaedic journals, two from general journals, two from infectious journals, and 1 from biomaterials journals, respectively. All of these journals are JCR Q1 journals and have great influence in their respective professional fields, for example, the New England Journal of Medicine. Among these top 10 cited articles, two were clinical guidelines in HPJI field, three were epidemiological investigations of HPJI (two were about the economic burden of HPJI, one is about the incidence of HPJI), the remaining 5 articles were evenly distributed in the 5 fields of new biomaterials research, standard use of antibiotics, analysis of risk factors and prognosis, new diagnostic methods, and clinical case discussion, with 1 article respectively. The focus point of each article was summarized in Table 1.

**Table 1:** Analysis of the top 10 cited articles in HPJI field.

Rank	Article	Journal	IF	JCR	Citations	Focus point
1	Osmon et al. (16)	Clin Infect Dis	9.079	Q1	1,236	Clinical practice guidelines for diagnosis and management of PJI
2	Bozic et al. (17)	J Bone Joint Surg Am	5.284	Q1	980	Infection is the third leading cause of revision THA in USA
3	Kurtz et al. (18)	J Arthroplasty	4.757	Q1	911	The economic burden of PJI will increase as the demand for joint arthroplasty increases
4	Trampuz et al. (19)	N Engl J Med	91.245	Q1	835	Culture of samples obtained by sonication of prostheses was more sensitive than conventional periprosthetic-tissue culture for the microbiologic diagnosis
5	Alt et al. (20)	Biomaterials	12.479	Q1	686	NanoSilver bone cement completely inhibited the proliferation of multiresistant bacteria in the absence of *in vitro* cytotoxicity
6	Kurtz et al. (21)	J Arthroplasty	4.757	Q1	622	Infection Burden for Hip and Knee Arthroplasty is increasing in USA
7	Bratzler et al. (22)	Clin Infect Dis	9.079	Q1	596	Provided an overview of other issues related to antimicrobial prophylaxis, including specific suggestions regarding antimicrobial selection.
8	Mahomed et al. (23)	J Bone Joint Surg Am	5.284	Q1	534	Factors associated with an increased risk of an adverse outcome included increased age, gender, race, a medical comorbidity, and a low income.
9	Del Pozo et al. (24)	N Engl J Med	91.245	Q1	512	Expounded how to standardize the clinical diagnosis and treatment of PJI through a specific case
10	Parvizi et al. (25)	J Arthroplasty	4.757	Q1	463	Expounded the d Definition of Periprosthetic Hip and Knee Infection by an evidence-based and validated criteria

IF, impact factor; JCR, journal citation reports; PJI, prosthetic joint infection; THA, total hip arthroplasty; TKA, total knee arthroplasty. The IF and JCR were based on the 2021 version.

### Hot spot analysis of the key field about HPJI

**Figure 7:**
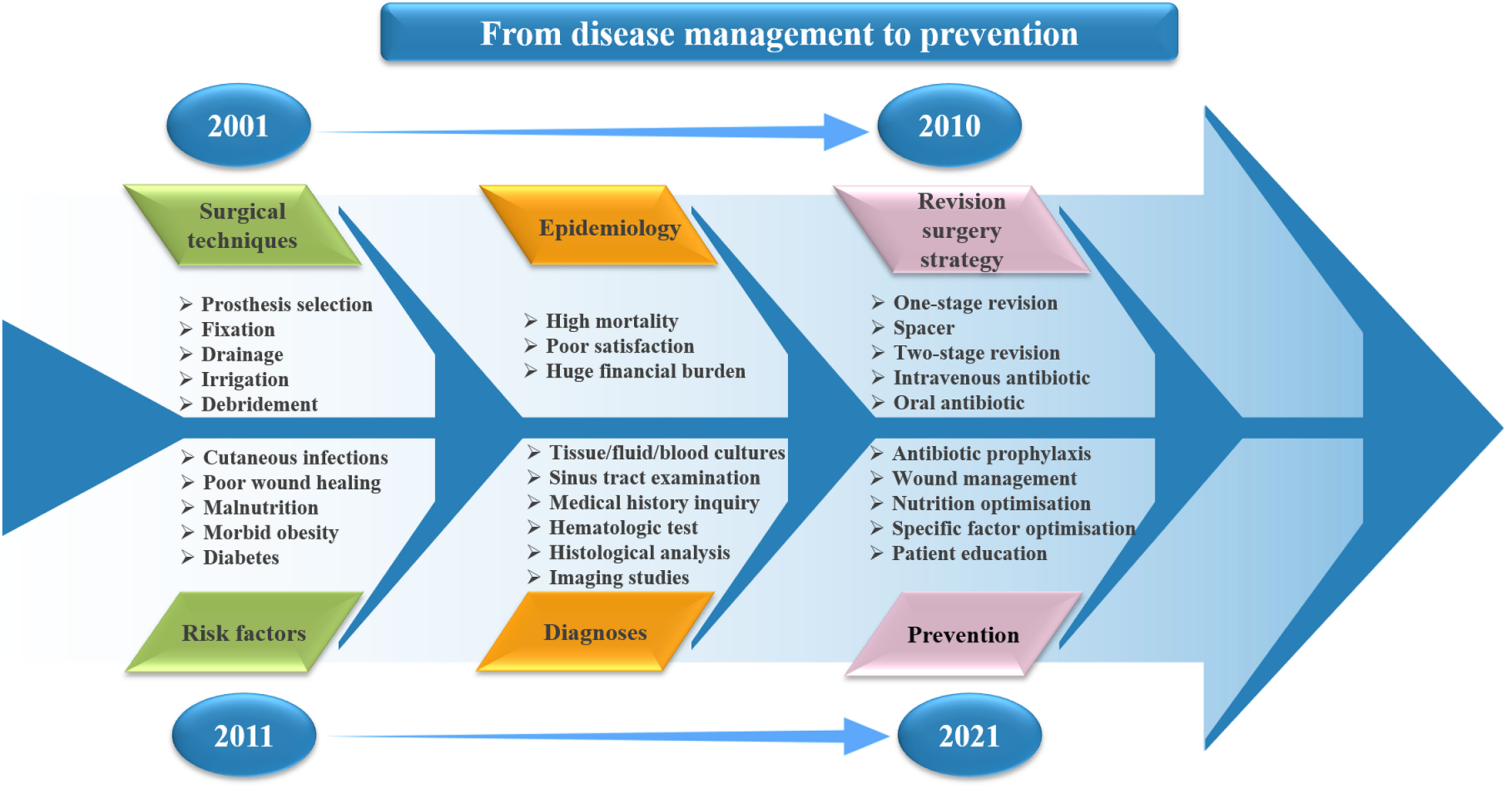
Hot spot analysis of the key field about HPJI. The fishbone diagram showed the research trends in the key field about HPJI. From 2001 to 2010, research hotspots in HPJI field were concentrated in surgical techniques, epidemiology, revision surgery strategy; From 2011 to 2021, the focus of research began to turn to risk factors, diagnoses and prevention. By summarizing the topics of papers published in different time periods, it can be concluded that the research trends in the HPJI field over the past 20 years are: From disease management to prevention.

Based on the results of co-occurrence analysis and the top 10 cited articles, we analyzed the hot spots in key field of HPJI (Figure 7). In surgical techniques field, debridement, irrigation, drainage, fixation and prosthesis selection were considered the most critical techniques. As for the risk factors, cutaneous infections, poor wound healing, malnutrition, morbid obesity and diabetes were the common but troubling factors (26). With regard to revision surgery strategy, one-stage revision, spacer, two-stage revision and intravenous/oral antibiotic application were the major concern for researchers. High mortality, poor satisfaction and huge financial burden were the main epidemiological characteristics of HPJI (17, 18). As for the diagnoses, it is suggested to complete the following six aspects to ensure accurate diagnosis: Tissue/fluid/blood cultures, sinus tract examination, hematologic test, histological analysis, imaging studies, medical history inquiry (24, 26). In the prevention of HPJI, antibiotic prophylaxis, wound management, nutrition optimization, specific factor optimization and patient education were considered the critical procedures. Combined with the time nodes of different keywords, the research trend of HPJI field can be summarized as followed: from disease management to prevention.

## Discussion

Bibliometric is an interdisciplinary tool that helps us understand the developments and hot topics in a discipline/research area (11, 12). With the assist of bibliometric, we sorted out what could be focused on in HPJI field from aspects of the number of publications, contributions and cooperation of different authors, institutions, countries and journals, the research hotspot, and the top 10 cited articles.

In terms of the global research trend in HPJI field, over the past 20 years, HPJI has been studied multidimensionally and the number of publications been on the rise. The number of publications finally included in this study was 5,477, which means an average of nearly 274 publications per year. From 2001 to 2011, the number of publications published per year was below 200, while from 2012 to 2021, this number was exceeded 200. This means that research in the HPJI field has become more active since 2011.

We also clarified the most contributed authors/institutions/countries/journals in HPJI field. Parvizi J (Rothman Institute, Thomas Jefferson University, Philadelphia, PA, USA), with the largest number of publications and highest H-index, is the most contributed author in HPJI field. Rothman institute has the largest number of publications and highest H-index, followed was Jefferson University. Although Jefferson University (now named Thomas Jefferson University) did not have the largest number of publications or H-index, considering that both Rothman institute and Parvizi J are affiliated to this university, it is therefore arguably the most influential institution in the HPJI field. The USA had the largest number of publications and highest H-index. Additionally, Journal of Arthroplasty (with the largest number of publications) and Journal of Bone and Joint Surgery American Volume (with the highest H-index) were both founded in the USA. Although the USA has contributed most in this field, co-analysis and bibliographic coupling analysis suggest that authors, institutions and journals from other countries (e.g., Germany, the UK, Canada and China) are in close contact with the United States, and their publications and contribution are increasing. Since HPJI is a difficult disease to treat, ascendent collaboration would be beneficial for further research. For example, Yang J et al. initiated a multicentre randomized controlled trial, indicated that after a two-stage revision of total hip or knee arthroplasty for chronic PJI, a three-month course of microorganism-directed, oral antibiotics could significantly reduce the rate of failure due to further infection (27).

As for the co-occurrence analysis, from a macro perspective, surgical techniques, risk factors, revision surgery strategy, epidemiology, diagnoses and prevention were the hot research direction of HPJI field. From a micro perspective, replacement, total hip arthroplasty, infection, arthroplasty and total hip were the hot keywords. From the change of keywords in different time, most studies focused on hip replacement techniques and infection management from 2001 to 2010. From 2010 to 2021, risk factors analysis, novel diagnostic techniques and indicators became more prominent in HPJI field. This indicates that the concentration of HPJI has changed over the past 20 years, shifting from the technique of THA and management of infection to the risk factors analysis, and new diagnostic methods and techniques. This reflects that more attention has been paid to the prevention of HPJI. As a recent 15years population-based cohort study showed that male sex, type-2 diabetes mellitus, and discharge to convalescent care were associated with an increased risk of HPJI, the prevention of HPJI after THA deserve more attention (28). Additionally, from the 2013 version of the clinical guidelines (16), 2016 review published in the Lancet (26), to the 2018 version of the definition of PJI (25), the importance of diagnosis was emphasized, which suggests that we should also focus on the diagnosis of HPJI.

In terms of the top 10 cited articles, two were clinical guidelines in HPJI field, three were epidemiological investigations of HPJI (two were about the economic burden of HPJI, one is about the incidence of HPJI), the remaining 5 articles were equally distributed in the 5 fields, new biomaterials research, standard use of antibiotics, analysis of risk factors and prognosis, new diagnostic methods, and clinical case discussion namely. The research directions of these top 10 cited articles were basically consistent with the results of co-occurrence analysis, which once again underscores the importance of keeping track of research trends and priorities. More importantly, the number of citations reflects the high research value of these articles (29), an intensive reading of these articles will help us to understand and master the trend and focus of HPJI research comprehensively.

Based on the results of co-occurrence analysis and the top 10 cited articles, we also analyzed the hot spots in key field of HPJI. Combined with the time nodes of different keywords, the research trend of HPJI field can be summarized as followed: from disease management to prevention. The top 10 cited articles about HPJI gave us the enlightenments that: Timely disease management after the occurrence of HPJI can help solve the problems of patients, but it will inevitably bring huge economic burden, while patients also need to bear certain medical risks. Therefore, a better solution for HPJI is to improve the efficiency of prevention, new surgical techniques and preventive protocols should be taken seriously. Scholars have made efforts in this field, specifically as follows: Identifying high risk patients for HPJI is a key step in preventing it from happening, so patient-specific risk optimization is of great significance. Perioperative antibiotic usage, such as dual antibiotic therapy for preoperative administration, single vs. multiple doses of preoperative antibiotics, the use of local antibiotic treatment such as vancomycin powder (30).

There are some limitations in this study, which is the goal of our efforts to improve in the future. First, due to limitations of software and analytical methods, the data for our study were only from WOSCC. Second, all the literatures included in this study were in English, and high-quality literatures in other languages, such as Chinese, were not included in the analysis. It is worth noting that with the improvement of the literature evaluation system and the progress of AI, it is expected to solve the problems of literature citation and self-citation in the future, and the h index and impact factor system are not objective enough, so as to further enhance the positive role of bibliometrics in the development of the discipline. Furthermore, the medico-legal implications in the context of HPJI are highly significant and cannot be overlooked, given the substantial impact these complications have on both patient health and the responsibilities of healthcare professionals. This importance runs through the management and prevention of HPJI, in the comprehensive management of primary hip replacement and HPJI, doing every detail of diagnosis, treatment, prevention, follow-up and other areas is the key to avoid causing medical legal disputes/problems (31).

## Conclusion

This study showed a bibliometric analysis of the global research trends and top 10 cited articles in HPJI field. We reported that HPJI has been researched multidimensionally and the number of publications was rising in the past 20 years. The USA has made outstanding contributions, and the exchanges and cooperation among countries in HPJI field around the world were active. Surgical techniques, risk factors and outcomes, revision surgery strategy, diagnoses, causes of infection and epidemiology were the hot research direction of HPJI, and the focus of hotspots had shifted from disease management to prevention. The core content of the top 10 cited articles is basically consistent with the above research direction. Intensive reading of these articles may help us to understand the trend and focus of HPJI research comprehensively.

## Data Availability

The original contributions presented in the study are included in the article/Supplementary Material, further inquiries can be directed to the corresponding author.
